# ZNF280A and ACRV1 enhance aerobic glycolysis and drive ovarian cancer progression *via* the PI3K/AKT signaling pathway

**DOI:** 10.1016/j.jbc.2025.110993

**Published:** 2025-12-01

**Authors:** Dawei Zhu, Puyu Chen, Liangbin Yu, Shuai Gao, Yi Liu, Silin Pan, Li Li

**Affiliations:** 1 Heart Center, Women and Children's Hospital, Qingdao University, Qingdao, P. R. China; 2 Department of Gynaecology and Obstetrics, Daping Hospital, Army Medical University, Chongqing, P. R. China

**Keywords:** OC, ZNF280A, ACRV1, proliferation, apoptosis, migration

## Abstract

Ovarian cancer (OC) remains a leading cause of gynecological cancer–related mortality, largely due to metabolic reprogramming and aggressive progression. Zinc finger protein 280A (ZNF280A), a poorly characterized transcriptional regulator, has recently been implicated in tumorigenesis, but its mechanistic role in OC remains undefined. Here, we identify ZNF280A as an oncogenic driver that promotes OC progression through transcriptional regulation of acrosomal vesicle protein 1 (ACRV1) and activation of the PI3K/AKT signaling pathway. ZNF280A expression was markedly elevated in OC tissues and cell lines and correlated with advanced clinicopathologic features and poor patient survival. Functional assays revealed that ZNF280A knockdown inhibited OC cell proliferation, migration, and tumorigenesis while inducing apoptosis both *in vitro* and *in vivo*. Mechanistically, ZNF280A enhanced *ACRV1* transcription by interacting with the transcription factor CUX2, thereby facilitating its recruitment to the *ACRV1* promoter. Elevated ZNF280A or ACRV1 expression activated PI3K/AKT signaling and increased glycolytic enzyme expression (PKM2 and LDHA), glucose uptake, lactate production, ATP generation, and extracellular acidification rate, whereas pharmacological inhibition of AKT or glycolysis abrogated these effects. Collectively, our findings establish ZNF280A as a key regulator of metabolic reprogramming in OC through the CUX2–ACRV1–PI3K/AKT axis, highlighting this pathway as a potential therapeutic target in ovarian cancer.

Ovarian cancer (OC) is one of the most lethal gynecological malignancies, characterized by insidious onset, rapid progression, and poor prognosis (1). Despite advances in cancer prevention and therapy, the 5-year survival rate for OC remains low, largely due to late-stage diagnosis and frequent recurrence (2). Consequently, the development of novel therapeutic strategies to improve patient outcomes remains a critical research priority.

Accumulating evidence highlights metabolic reprogramming, particularly aerobic glycolysis (the Warburg effect), as a hallmark of cancer progression and chemoresistance. Unlike normal cells that rely on oxidative phosphorylation, tumor cells preferentially convert glucose to lactate even under normoxic conditions, thereby sustaining proliferation and survival. Oncogenic signaling pathways such as PI3K/AKT/mTOR, HIF-1α, and MYC orchestrate this metabolic shift by upregulating glycolytic enzymes and glucose transporters. In OC, enhanced glycolysis not only fuels tumor growth but also remodels the microenvironment to promote invasion, immune evasion, and drug resistance (3, 4, 5, 6, 7). Thus, targeting aerobic glycolysis offers a promising avenue for therapeutic intervention in OC.

Zinc finger protein 280A (ZNF280A) belongs to the zinc finger protein family, which mediates DNA/RNA binding and regulates gene transcription and chromatin remodeling (8). Emerging evidence indicates that ZNF280A plays multifaceted roles in tumorigenesis (9). Although its role in OC remains unclear, findings from other cancers provide valuable clues. ZNF280A dysregulation has been linked to tumor progression and metastasis in bladder cancer (10), regulation of cell cycle and apoptosis in lung adenocarcinoma (11), and activation of the PI3K/AKT signaling pathway in colorectal cancer (12). These observations suggest that ZNF280A may act as an oncogenic driver, and investigating its involvement in the aerobic glycolysis pathway could be key to understanding OC progression.

## Results

### The correlation between ZNF280A expression and OC

**Figure 1:**
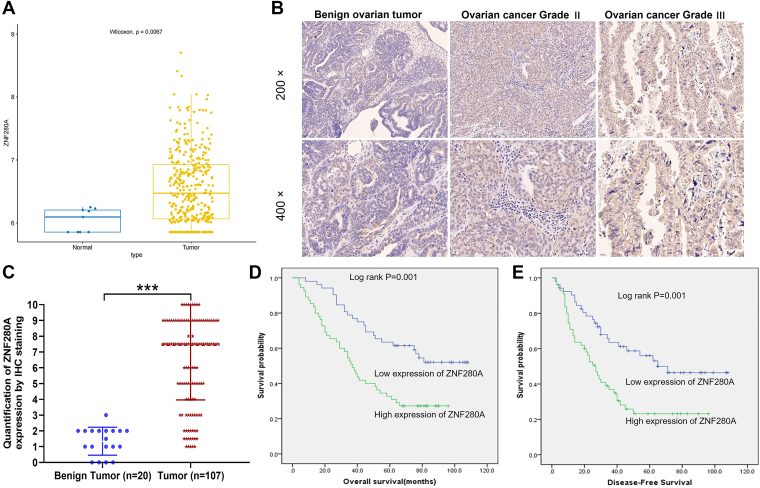
**Elevated ZNF280A expression correlates with poor survival outcomes in OC**. *A*, analysis of *ZNF280A* expression in OC and adjacent normal tissues utilizing RNA sequencing transcript data from the Cancer Genome Atlas ovarian cancer (TCGA-OV) cohort. *B*, IHC staining assays were performed in ovarian cancer to reveal the protein expression of ZNF280A. *C*, quantitative IHC staining of tumor tissues and benign tumor tissues are presented to confirm the expression of ZNF280A. *D*–*E*, the clinical significance of ZNF280A expression and overall survival or disease-free survival based on Kaplan–Meier analysis using clinical OC patient’s follow-up data. IHC, immunohistochemical; OC, ovarian cancer.

To investigate the expression pattern of ZNF280A in OC, we analyzed RNA sequencing data from The Cancer Genome Atlas Ovarian Cancer (TCGA-OV) cohort. The results revealed a significant upregulation of *ZNF280A* in tumor tissues compared with adjacent normal ovarian tissues (*p* < 0.001) (Fig. 1*A*), suggesting a potential role in OC progression. To further validate the database findings, we performed immunohistochemical (IHC) staining on clinical OC samples. Representative IHC images showed markedly stronger ZNF280A staining in ovarian cancer tissues than in benign ovarian tumor tissues (Fig. 1*B*). Quantitative analysis indicated that 51.4% (55/107) of tumor tissues exhibited high ZNF280A expression, which was significantly higher than that in benign ovarian tumor tissues (*p* < 0.001) (Fig. 1*C*, Table 1).

**Table 1:** Expression patterns in ovarian cancer tissues and normal ovarian tissues was revealed by immunohistochemistry analysis

ZNF280A expression	Tumor tissue		Normal ovarian tissue		*p* value
	Cases	Percentage	Cases	Percentage	
Low	52	48.6%	19	100%	*p* < 0.001
High	55	51.4%	0	0%	

Furthermore, we employed Mann–Whitney U and Spearman correlation coefficient analyses to explore the associations between ZNF280A expression and clinicopathological characteristics of OC patients. Our findings uncovered significant positive correlations between ZNF280A expression and clinical grade (*p* < 0.001), tumor size (*p* < 0.001), and tumor-node-metastasis staging (*p* < 0.05) (Tables 2 and 3), implying that ZNF280A upregulation parallels disease progression. Importantly, Kaplan–Meier survival analysis reinforced the prognostic significance of ZNF280A, revealing that patients with high ZNF280A expression had significantly shorter survival times (Fig. 1, *D* and *E*). Collectively, these results underscore the potential of ZNF280A as a prognostic biomarker in OC, with its increased expression intimately linked to the malignancy of the tumor.

**Table 2:** Relationship between ZNF280A expression and tumor characteristics in patients with ovarian cancer

Features	Patients number	ZNF280A expression		*p* value
		low	High	
All patients	107	52	55	
Age (years)				0.771
<52	53	25	28	
≥52	54	27	27	
Grade				*p* < 0.001
I	12	11	1	
II	7	6	1	
III	88	35	53	
Stage				0.013
1	2	2	0	
2	22	14	8	
3	53	26	27	
4	30	10	20	
Tumor size				*p* < 0.001
<13.8 cm	53	35	18	
≥13.8 cm	54	17	37	
T Infiltrate (T)				0.039
T1	2	2	0	
T2	22	14	8	
T3	83	36	47	
Lymphatic metastasis (N)				0.040
N0	72	40	32	
N1	35	12	23	
Metastasis (M)				0.050
M0	77	42	35	
M1	30	10	20	
Recurrence of state				0.322
No	13	8	5	
Yes	94	44	50	
HOvaC1541Su01 KI67				0.351
<1.75	44	19	25	
≥1.75	63	33	30	
HOvaC1541Su01 EGFR				0.976
≤0.5	66	32	34	
>0.5	41	20	21	
HOvaC1541Su01 PDL1				0.147
<0.5	32	19	13	
≥0.5	75	33	42

**Table 3:** Relationship between ZNF280A expression and tumor characteristics in patients with ovarian cancer

Clinicopathological characteristics	Statistical parameter	ZNF2800 A
Grade	Spearman correlation coefficient	0.381
	Significance (double tail)	*p* < 0.001
	N	107
Stage	Spearman correlation coefficient	0.240
	Significance (double tail)	0.013
	N	107
T Infiltrate	Spearman correlation coefficient	0.201
(T)	Significance (double tail)	0.038
	N	107
Lymphatic metastasis	Spearman correlation coefficient	0.200
(N)	Significance (double tail)	0.039
	N	107
Metastasis	Spearman correlation coefficient	0.191
(M)	Significance (double tail)	0.049
	N	107
Tumor size	Spearman correlation coefficient	0.346
	Significance (double tail)	*p* < 0.001
	N	107

### ZNF280A knockdown inhibits proliferation, migration, and enhances apoptosis in OC cells

**Figure 2:**
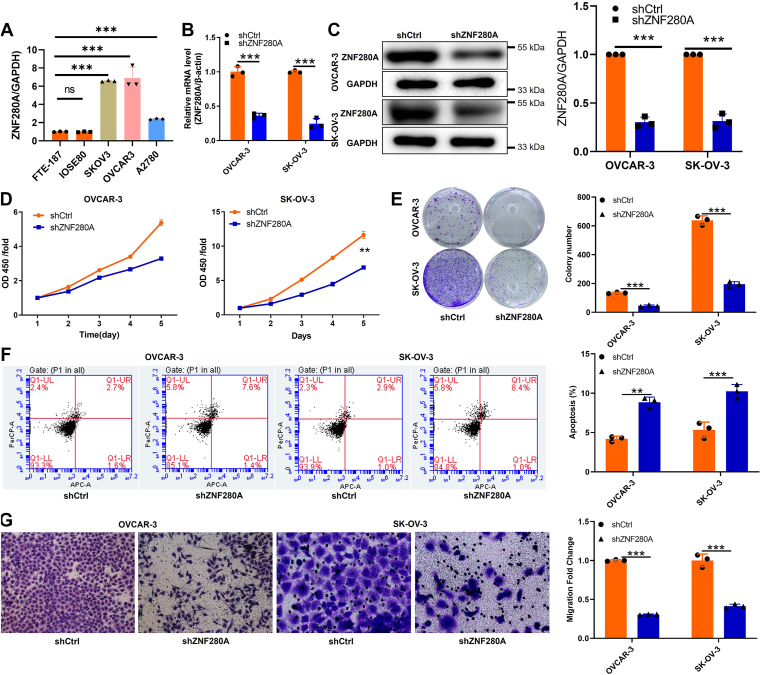
**Knockdown of ZNF280A inhibits cell proliferation, migration, and promotes apoptosis in OC cells**. *A*, the mRNA levels of *ZNF280A* in normal ovarian epithelial cells IOSE80, FTE-187, and OC cell lines A2780, OVCAR-3 SK-OV-3 were detected by qRT-PCR. *B*–*C*, after shZNF280A and shCtrl sequences were transfected into OVCAR-3 and SK-OV-3 cells, the expression of ZNF280A was detected by (*B*) qRT-PCR and (*C*) WB. *D*, cell proliferation of OVCAR-3 and SK-OV-3 cells with or without knockdown of ZNF280A is evaluated by CCK-8 assay. *E*, the number of cell colony in OVCAR-3 and SK-OV-3 cells with or without knockdown of ZNF280A is evaluated by colony formation assay. *F*, flow cytometry analysis based on Annexin V-APC/PI staining is utilized to detect the percentage of early apoptotic cell for OVCAR-3 and SK-OV-3 cells. *G*, cell migration of OVCAR-3 and SK-OV-3 cells with or without knockdown of ZNF280A is evaluated by Transwell assay. The data are expressed as mean ± SD (n = 3), ∗*p* < 0.05, ∗∗*p* < 0.01, ∗∗∗*p* < 0.001. CCK-8, Cell Counting Kit-8; OC, ovarian cancer; qRT-PCR, quantitative real-time-PCR; WB, western blot.

To investigate the functional consequences of ZNF280A knockdown in OC cells, we first confirmed its elevated mRNA expression in OC cell lines (A2780, OVCAR-3, and SK-OV-3) compared to normal ovarian epithelial cells (IOSE80 and FTE-187) (*p* < 0.001) (Fig. 2*A*). Subsequently, we targeted the highly expressing OVCAR-3 and SK-OV-3 cells with shRNA-mediated *ZNF280A* knockdown, achieving a significant reduction in both mRNA (87.7% and 59.1%, respectively; *p* < 0.001) (Fig. 2*B*) and protein levels (Fig. 2*C*), thereby establishing stable ZNF280A knockdown models. Functional assessments using Cell Counting Kit-8 (CCK-8) assay and colony formation assays revealed that ZNF280A silencing markedly inhibited cell proliferation in both OVCAR-3 and SK-OV-3 cells (*p* < 0.01 and *p* < 0.001, respectively) (Fig. 2, *D* and *E*). In addition, apoptotic cell ratios significantly increased in the ZNF280A-knockdown groups (*p* < 0.01), indicating proapoptotic effects (Fig. 2*F*). Furthermore, Transwell migration assays demonstrated a substantial decrease in the number of migrated cells post-ZNF280A knockdown, suggesting an inhibitory role in cell migration (*p* < 0.001) (Fig. 2*G*). Collectively, these findings underscore the pivotal role of ZNF280A in promoting OC cell proliferation and migration while suppressing apoptosis.

### Regulation mechanism of ZNF280A and ACRV1 in OC

**Figure 3:**
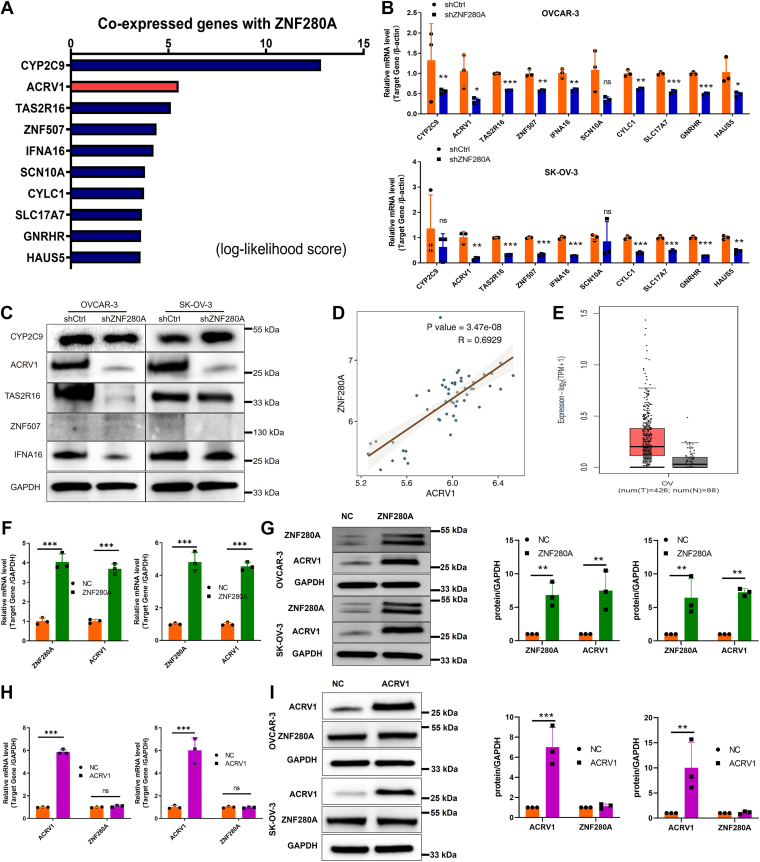
**Regulation mechanism of ZNF280A and ACRV1 in OC**. *A*, gene coexpression analysis was performed based on GEO database, and multiple genes coexpressed with *ZNF280A* were identified, such as *ACRV1*. *B*–*C*, the effect of ZNF280A knockdown on the expression of coexpressed genes was detected by qRT-PCR and WB. *D*, ZNF280A and ACRV1 expression level correlation analysis was conducted using TCGA-OV patient data. *E*, analysis of ACRV1 expression in OC and adjacent normal tissues utilizing RNA sequencing transcript data from the TCGA-OV cohort. *F*–*G*, overexpression of ZNF280A markedly increased *ACRV1* mRNA and protein expression levels. *H*–*I*, in contrast, ACRV1 overexpression did not significantly alter *ZNF280A* mRNA or protein expression levels. The data are expressed as mean ± SD (n = 3), ∗*p* < 0.05, ∗∗*p* < 0.01, ∗∗∗*p* < 0.001. ACRV1, acrosomal vesicle protein 1; GEO, Gene Expression Omnibus; OC, ovarian cancer; qRT-PCR, quantitative real-time-PCR; TCGA-OV, The Cancer Genome Atlas Ovarian Cancer; WB, western blot.

To further delineate the regulatory network involving ZNF280A in OC, we conducted comprehensive gene coexpression analysis leveraging the Gene Expression Omnibus (GEO) database, which identified acrosomal vesicle protein 1 (*ACRV1*) as a prominent coexpression partner of ZNF280A (Fig. 3*A*). Moreover, ZNF280A knockdown can downregulate the expression of ACRV1 (Fig. 3, *B* and *C*). In addition, TCGA database analysis reveals a significant positive correlation between *ACRV1* and *ZNF280A* expression levels (Fig. 3*D*), underscoring their functional interplay. Notably, TCGA database analysis illuminated *ACRV1*'s aberrant upregulation across multiple cancer types, particularly in tumors compared to normal tissues (Figs. 3*E* & S1*A*). Furthermore, overexpression of ZNF280A in OVCAR-3 and SK-OV-3 cells significantly enhanced *ACRV1* transcript abundance (Fig. 3, *F* and *G*), whereas ACRV1 overexpression did not exert a reciprocal effect on ZNF280A expression (Fig. 3, *H* and *I*). Collectively, these findings suggest that ZNF280A may regulate ovarian cancer progression through its downstream target gene *ACRV1*.

### ZNF280A promotes *ACRV1* transcription through CUX2

**Figure 4:**
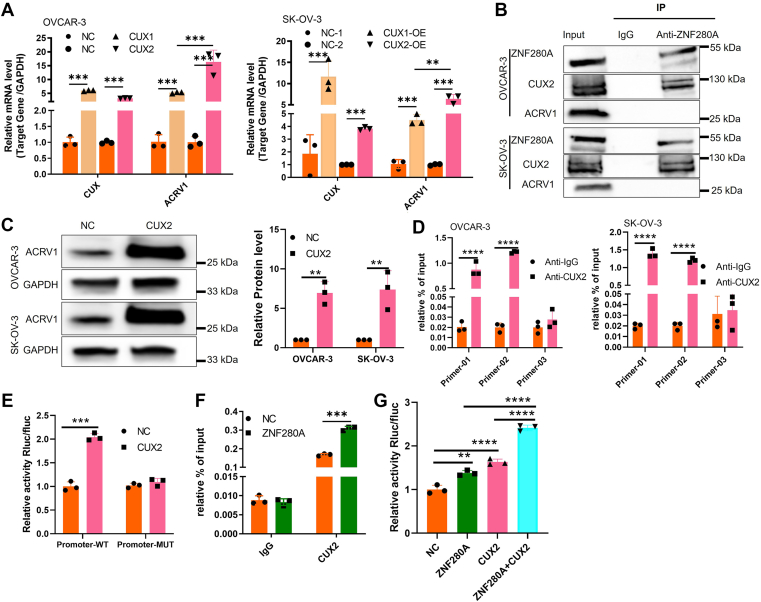
**ZNF280A promotes ACRV1 transcription through CUX2**. *A*, qRT-PCR analysis of *CUX1*, *CUX2*, and *ACRV1* mRNA levels in OVCAR-3 and SK-OV-3 cells following overexpression of CUX1 or CUX2. *B*, co-immunoprecipitation (Co-IP) assays confirmed the physical interaction between ZNF280A and CUX2 in OVCAR-3 and SK-OV-3 cells. *C*, western blot analysis showing that CUX2 overexpression markedly elevated ACRV1 protein levels in both OVCAR-3 and SK-OV-3 cells. *D*, chromatin immunoprecipitation followed by qPCR (ChIP-qPCR) demonstrated the direct binding of CUX2 to the ACRV1 promoter region using three distinct primer sets. *E*, luciferase reporter assays verified that CUX2 transcriptionally activates ACRV1 expression, with a significant increase in promoter activity observed in the wild-type (WT) ACRV1 promoter compared with the mutant (MUT) construct. *F*, ChIP-qPCR analysis showed that ZNF280A overexpression enhanced the recruitment of CUX2 to the ACRV1 promoter. *G*, luciferase reporter assays demonstrated that coexpression of ZNF280A and CUX2 synergistically enhanced ACRV1 promoter activity compared with either alone. Data are presented as mean ± SD (n = 3). ∗*p* < 0.05, ∗∗*p* < 0.01, ∗∗∗*p* < 0.001, ∗∗∗∗*p* < 0.0001. ACRV1, acrosomal vesicle protein 1; qRT-PCR, quantitative real-time-PCR.

To further elucidate the mechanism by which ZNF280A regulates ACRV1 transcription, we conducted a series of mechanistic investigations. Bioinformatic prediction revealed that ZNF280A itself is not a direct transcription factor of ACRV1. Interestingly, protein–protein interaction analysis suggested that ZNF280A may interact with CUX1 and CUX2, two predicted transcription factors of ACRV1. To verify this, we overexpressed CUX1 and CUX2 in OVCAR-3 and SK-OV-3 cells, respectively, and found that CUX2 overexpression more effectively enhanced ACRV1 transcription (Fig. 4*A*). Furthermore, co-immunoprecipitation (Co-IP) assays confirmed the physical interaction between ZNF280A and CUX2 (Fig. 4*B*). Overexpression of CUX2 markedly elevated ACRV1 protein levels (Fig. 4*C*), and chromatin immunoprecipitation followed by qPCR (ChIP-qPCR) demonstrated the direct binding of CUX2 to the ACRV1 promoter region (Fig. 4*D*). Luciferase reporter assays further verified that CUX2 transcriptionally activates ACRV1 expression (Fig. 4*E*), confirming that CUX2 functions as a transcription factor for ACRV1. Moreover, ChIP-qPCR assays revealed that ZNF280A overexpression promoted the recruitment of CUX2 to the ACRV1 promoter (Fig. 4*E*), while luciferase assays showed that ZNF280A overexpression significantly enhanced CUX2-mediated transcriptional activation of ACRV1 (Fig. 4*F*). Collectively, these findings demonstrate that ZNF280A facilitates ACRV1 transcription by enhancing CUX2 binding and transcriptional activity at the ACRV1 promoter.

### ZNF280A and ACRV1: a novel regulatory axis driving OC cell proliferation, migration, and apoptosis resistance

**Figure 5:**
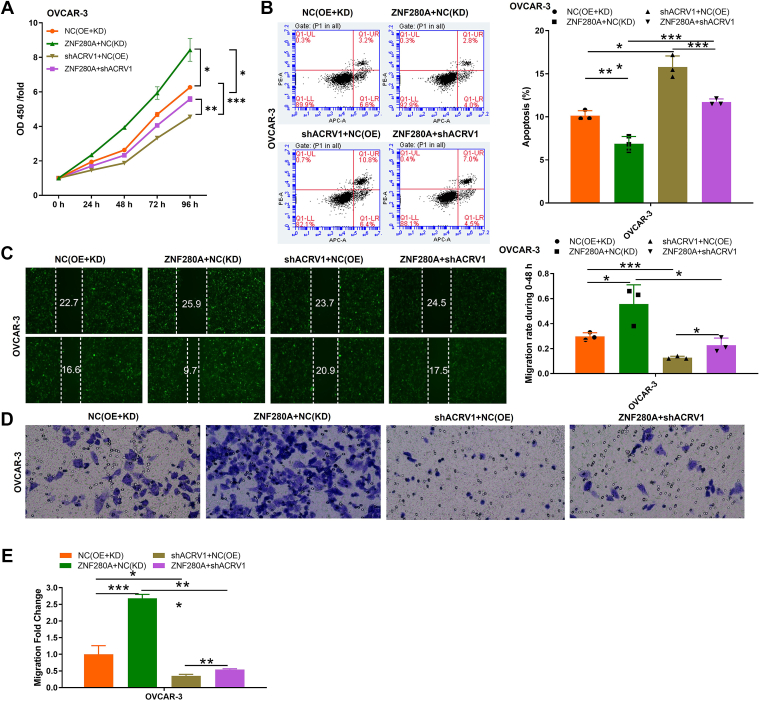
**ZNF280A and ACRV1 driving OC cell proliferation, migration, and apoptosis resistance**. *A*–*E*, after constructing ZNF280A overexpression and ACRV1 knockdown in OVCAR-3 cells, phenotypic tests were performed, such as (*A*) proliferation, (*B*) apoptosis, and (*C*–*E*) migration. The data are expressed as mean ± SD (n = 3), ∗*p* < 0.05, ∗∗*p* < 0.01, ∗∗∗*p* < 0.001. ACRV1, acrosomal vesicle protein 1; OC, ovarian cancer.

To determine whether ZNF280A regulates cellular functions through ACRV1, we manipulated ACRV1 and ZNF280A expression in OC cells (Fig. S1, *B*–*D*). Importantly, while ZNF280A overexpression potently enhanced OC cell proliferation (OVCAR-3) (*p* < 0.01), this effect was significantly attenuated by ACRV1 knockdown (*p* < 0.001) as evidenced by CCK-8 assays (Fig. 5*A*). Furthermore, flow cytometry analysis revealed that ACRV1 knockdown partially reversed the antiapoptotic effect of ZNF280A overexpression in OC cells (*p* < 0.001) (Fig. 5*B*). Similar trends were observed in migration assays, where ZNF280A overexpression facilitated OC cell migration, whereas ACRV1 knockdown mitigated this phenotype (*p* < 0.001) as determined by Transwell and wound healing assays (Fig. 5, *C*–*E*). Collectively, these findings underscore the pivotal role of ACRV1 in mediating ZNF280A's regulatory effects on OC cell proliferation, migration, and apoptosis, thereby revealing a novel therapeutic target in OC pathogenesis.

### ZNF280A and ACRV1 regulate aerobic glycolysis and drive OC cell progression through PI3K/AKT signaling pathway

**Figure 6:**
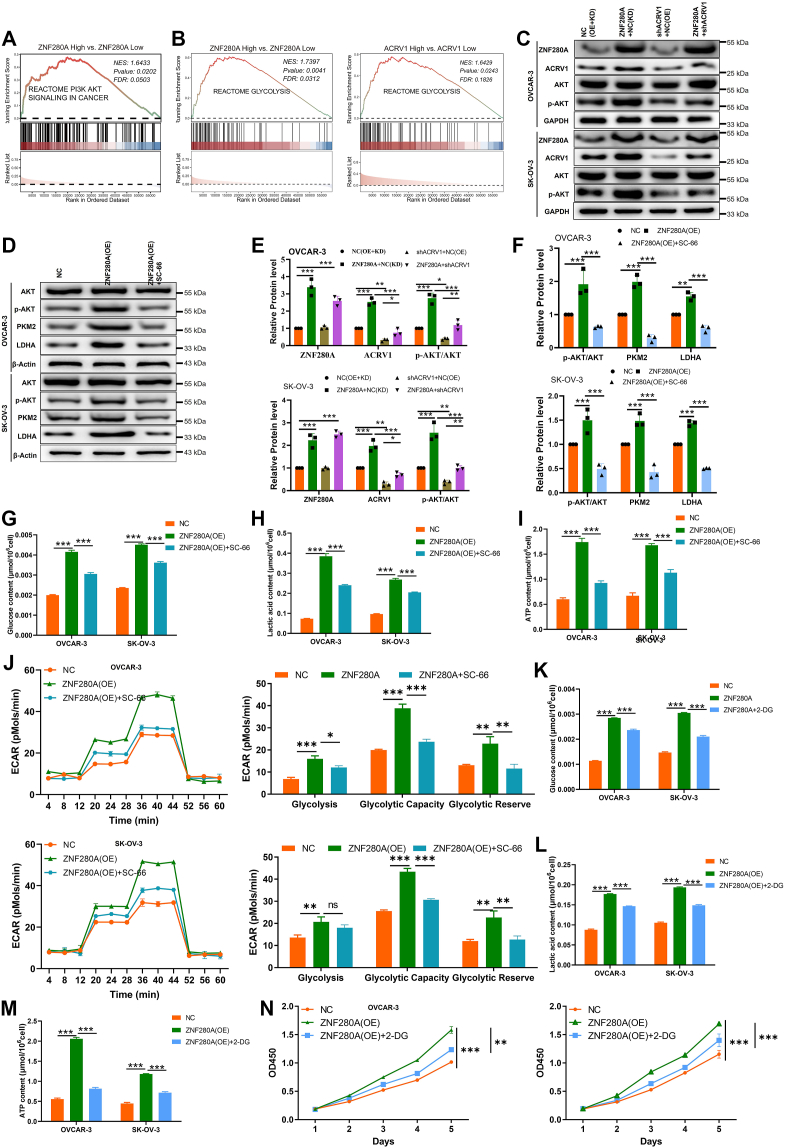
**ZNF280A and ACRV1 regulate aerobic glycolysis and drive OC cell progression through PI3K/AKT signaling pathway**. *A*–*B*, gene set enrichment analysis (GSEA) revealed that high ZNF280A expression was associated with the activation of the PI3K/AKT signaling pathway, whereas elevated expression of both ZNF280A and ACRV1 was linked to enrichment of glycolysis-related pathways. *C*, western blot analysis showing that ZNF280A overexpression upregulated ACRV1 and enhanced AKT phosphorylation, while ACRV1 knockdown reduced these effects in OVCAR-3 and SK-OV-3 cells. *D*, ZNF280A overexpression increased the expression of glycolytic enzymes PKM2 and LDHA in OC cells. *E*, quantitative analysis of ZNF280A, ACRV1, and p-AKT protein levels confirming that ACRV1 knockdown attenuated ZNF280A-induced AKT activation. *F*, inhibition of AKT signaling using SC-66 reversed the upregulation of PKM2 and LDHA caused by ZNF280A overexpression. *G*–*I*, ZNF280A overexpression significantly increased glucose uptake, lactate production, and intracellular ATP levels, whereas treatment with SC-66 suppressed these effects. *J*, extracellular acidification rate (ECAR) assays demonstrated enhanced glycolytic flux, glycolytic capacity, and glycolytic reserve upon ZNF280A overexpression, which were reversed by SC-66 treatment. *K*–*M*, treatment with the glycolysis inhibitor 2-deoxy-D-glucose (2-DG) reduced glucose uptake, lactate production, and ATP generation in ZNF280A-overexpressing OC cells. *N*, cell viability assays showed that 2-DG treatment markedly attenuated the proliferative advantage conferred by ZNF280A overexpression. Data are presented as mean ± SD (n = 3). ∗*p* < 0.05, ∗∗*p* < 0.01, ∗∗∗*p* < 0.001, ∗∗∗∗*p* < 0.0001. OC, ovarian cancer; ACRV1, acrosomal vesicle protein 1; ZNF280A, zinc finger protein 280A.

To elucidate how the ZNF280A/ACRV1 axis regulates ovarian cancer progression, we performed gene set enrichment analysis (GSEA). The results showed that high ZNF280A expression was significantly associated with the activation of the PI3K-AKT signaling pathway (Fig. 6*A*). In addition, elevated expression of both ZNF280A and ACRV1 markedly upregulated glycolysis-related pathways (Fig. 6*B*). Given the pivotal role of AKT in regulating glycolysis, we hypothesized that the ZNF280A/ACRV1 axis may modulate glycolytic activity through the AKT pathway. We further conducted experimental validation to confirm this hypothesis. Notably, ZNF280A overexpression facilitated the upregulation of ACRV1 and subsequently augmented AKT phosphorylation, whereas ACRV1 knockdown attenuated these effects (Fig. 6, *C* and *E* & Fig. S1*E*), hinting at a potential coordinated regulation of the PI3K/AKT pathway by ZNF280A and ACRV1.

Given the well-established role of the PI3K/AKT signaling pathway in modulating aerobic glycolysis, it becomes imperative to delve deeper into the potential involvement of ZNF280A in this metabolic process. Our observations demonstrated that the overexpression of ZNF280A in OC cells led to an upregulation of pivotal enzymes implicated in aerobic glycolysis, including PKM2 and LDHA (Fig. 6, *D* and *F*). This overexpression was also accompanied by enhancements in glucose uptake (Fig. 6*G*), lactic acid production (Fig. 6*H*), ATP content (Fig. 6*I*), and the extracellular acidification rate (Fig. 6*J*). Intriguingly, the treatment of OC cells overexpressing ZNF280A with the AKT inhibitor (SC-66) reversed these effects (Fig. 6, *G*–*J*), implying that ZNF280A may orchestrate aerobic glycolysis through the PI3K/AKT signaling pathway.

Furthermore, the exposure of OC cells overexpressing ZNF280A to 2-DG, an inhibitor of aerobic glycolysis, resulted in significant downregulation of glucose uptake, lactic acid production, and ATP content (Fig. 6, *K*–*M*). Notably, this treatment also markedly impaired cell viability (Fig. 6*N*). These cumulative findings underscore the pivotal role of ZNF280A in propelling abnormal proliferation of OC cells by potentiating aerobic glycolysis.

### ZNF280A knockdown attenuates the tumorigenic potential of OC *in vivo*

**Figure 7:**
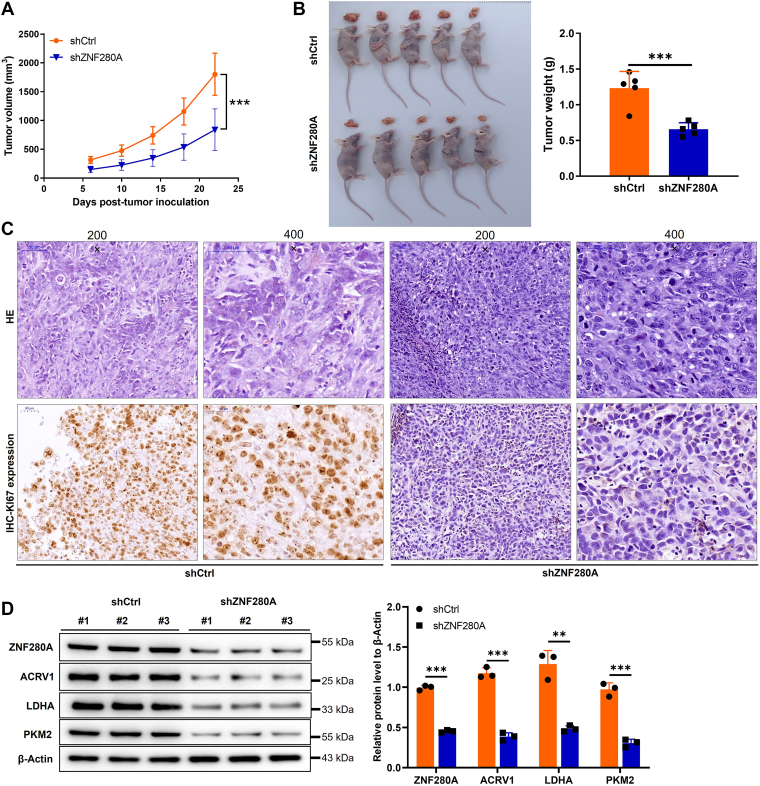
**Knockdown of ZNF280A inhibits tumor growth in mice xenograft models**. *A*, the volume of tumors in shCtrl group and shZNF280A group is measured after post injection. *B*, the tumors image and weight in shCtrl group and shZNF280A group. *C*, HE and IHC staining assays were performed in tumor tissues of mice to reveal the protein expression of KI67. *D*, the expression of key glycolytic enzymes was detected by WB in tumor tissues with ZNF280A knocked down and control. Data are presented as mean ± SD (n = 3). ∗*p* < 0.05, ∗∗*p* < 0.01, ∗∗∗*p* < 0.001. IHC, immunohistochemical; WB, western blot.

Here, we established the mouse xenograft models to determine the regulatory role of ZNF280A in OC. Our findings revealed a profound reduction in tumor volume in the shZNF280A-treated cohort, as evidenced by a statistically significant decrease (*p* < 0.001) compared to the shCtrl control group (Fig. 7*A*) Furthermore, the average tumor weight in the shZNF280A group was markedly diminished compared to the control group (*p* < 0.001) (Fig. 7*B*). To gain insights into the underlying mechanisms, we assessed HE Staining, tumor sections showed morphological features of high-grade serous ovarian cancer, including papillary structures and nuclear atypia (Fig. 7*C*). Notably, the IHC staining outcomes indicated a substantially lower level of the proliferation marker KI67 expression in the shZNF280A group, indicating a suppression of cellular proliferation, as compared to the shCtrl group (Fig. 7*C*). In addition, the downregulation of key glycolytic enzymes in tumor tissues following ZNF280A knockdown further corroborates the aforementioned findings (Fig. 7*D*). Collectively, these results underscore the ability of ZNF280A silencing to attenuate the tumorigenic potential of OC *in vivo*, reinforcing the previously reported *in vitro* observations.

## Discussion

Our study contributes to the growing body of literature investigating the intricate mechanisms underlying OC progression. While previous research has hinted at the involvement of ZNF280A in cancer progression (10, 11, 12), its specific role in OC and its modulation of aerobic glycolysis have remained largely unexplored. Our findings, which demonstrate the upregulation of ZNF280A in OC tissues and its association with poor survival outcomes, align with previous studies that have identified ZNF280A as a potential oncogene in various cancer types. However, our study uniquely highlights the functional interplay between ZNF280A and ACRV1, a less well-studied protein, in driving OC progression through the PI3K/AKT pathway. This novel insight expands the current understanding of OC biology and suggests new avenues for therapeutic intervention.

In addition, our study uncovered a previously unrecognized transcriptional mechanism linking ZNF280A to ACRV1 regulation. Bioinformatic and biochemical analyses revealed that ZNF280A enhances CUX2-mediated transcriptional activation of ACRV1, rather than directly binding to the ACRV1 promoter. Co-immunoprecipitation and ChIP–qPCR assays confirmed the interaction between ZNF280A and CUX2 and the enhanced recruitment of CUX2 to the ACRV1 promoter upon ZNF280A overexpression. This ZNF280A–CUX2–ACRV1 transcriptional axis establishes a new layer of regulation that connects transcriptional control with downstream PI3K/AKT pathway activation, offering mechanistic insight into how ZNF280A coordinates metabolic reprogramming in OC cells.

Recent investigations have underscored metabolic reprogramming as a key determinant of OC aggressiveness, indicating that activation of the PI3K/AKT/mTOR pathway enhances glycolytic flux and promotes chemoresistance in OC (13, 14, 15). Our findings extend these observations by identifying a previously unrecognized upstream regulatory axis, in which ZNF280A transcriptionally activates ACRV1 through interaction with CUX2, thereby establishing a direct link between transcriptional regulation and metabolic adaptation. Unlike earlier studies focusing on enzymatic regulators or signaling intermediates, our work reveals a transcription-based mechanism that integrates oncogenic signaling with glycolytic control. Moreover, consistent with prior reports in colorectal and lung cancers where ZNF280A promotes tumorigenesis *via* PI3K/AKT activation, our data demonstrate a conserved yet context-specific regulatory paradigm in OC, coupling metabolic remodeling to cell proliferation and survival.

Biologically, the ZNF280A/ACRV1 axis functions as a metabolic switch that enhances glycolytic capacity, enabling OC cells to adapt to hypoxia and nutrient stress within the tumor microenvironment. This metabolic advantage may contribute to tumor aggressiveness, immune evasion, and chemoresistance—hallmarks of advanced OC. From a therapeutic standpoint, targeting the ZNF280A/ACRV1 axis could simultaneously suppress oncogenic signaling and metabolic reprogramming. The reversal of glycolytic activation by AKT inhibition or glycolysis blockade observed in our experiments supports this dual-targeting strategy. Future investigations should evaluate whether ZNF280A or ACRV1 can serve as predictive biomarkers for therapeutic response or as potential metabolic vulnerabilities. Moreover, combining inhibitors of glycolysis or AKT signaling with existing treatments such as platinum-based chemotherapy or PARP inhibitors may represent a promising approach to overcome metabolic plasticity in OC.

ACRV1, also named as SP-10, is an evolutionarily conservative acrosomal protein, which was first identified in human sperm (16). ACRV1 protein is specific to male germ cells and is not expressed anywhere else in the body (17). Thus, the gene encoding the SP-10 protein ACRV1 is an outstanding model for understanding the transcriptional regulation of male germ cell-specific genes (18). At present, only a few studies have revealed ACRV1 as a transcription factor involved in tumor migration (19, 20). However, ACRV1, a relatively understudied protein in cancer, emerges as a key player in our study, positively regulating ZNF280A and contributing to OC progression.

In addition, ZNF280A and ACRV1 positively regulate each other, influencing OC progression *via* the PI3K/AKT pathway. The PI3K/AKT pathway, a well-established signaling cascade implicated in numerous cancers (21, 22, 23). This finding resonates with previous research that has linked PI3K/AKT activation to enhanced aerobic glycolysis, a metabolic hallmark of cancer cells (24). For instance, studies in OC (25), bladder cancer (26), breast cancer (27) and lung cancer (28) have demonstrated that PI3K/AKT signaling promotes the Warburg effect, wherein cancer cells preferentially utilize glycolysis for energy production even in the presence of oxygen. Our results, which demonstrate that blockade of PI3K/AKT or glycolysis mitigates the effects of ZNF280A overexpression, further support the notion that targeting this pathway could be an effective therapeutic strategy in OC.

Although our study provides valuable insights into the mechanistic role of ZNF280A and ACRV1 in OC progression, several limitations must be acknowledged. Firstly, the sample size of OC tissues and cell lines analyzed in our study was relatively small, limiting the generalizability of our findings. Secondly, our functional assays were conducted primarily *in vitro* and in animal models, and further validation in human clinical trials is essential to assess the therapeutic potential of targeting ZNF280A, ACRV1, or the PI3K/AKT pathway in OC patients. In addition, the precise molecular mechanisms underlying the interaction between ZNF280A and ACRV1, as well as their regulation of aerobic glycolysis, remain to be fully elucidated.

In conclusion, our study underscores the importance of ZNF280A and ACRV1 in enhancing aerobic glycolysis and driving OC cell progression through the PI3K/AKT signaling pathway. These findings not only expand the current understanding of OC biology but also identify potential therapeutic targets for this devastating disease. By targeting ZNF280A, ACRV1, or the PI3K/AKT pathway, we may be able to disrupt the metabolic reprogramming that sustains OC cell growth and survival. Future research should aim to validate our findings in larger cohorts, explore the underlying molecular mechanisms, and develop novel therapeutic strategies to improve the outcomes of OC patients.

## Experimental procedures

### Tissue procurement and cell cultivation

From November 2009 to March 2018, a total of 107 OC tumor tissues and 20 adjacent nontumorous tissues were collected from patients diagnosed with OC. The diagnosis was confirmed through comprehensive clinical and pathological evaluations. All specimens were immediately snap-frozen and stored in liquid nitrogen to preserve tissue integrity. Prior to participation, written informed consent was obtained from all patients for the use of their clinical data and tissue samples for research purposes.

All procedures involving human participants were conducted in accordance with the ethical standards of the Ethics Committee of Army Medical University and with the principles of the Declaration of Helsinki. The study protocol and consent procedures were reviewed and approved by the institutional ethics board.

Regarding cell culture, human normal ovarian epithelial cell lines, IOSE80 and OC-derived cell lines, A2780, OVCAR-3, and SK-OV-3, were procured from the reputable cell bank at the Shanghai Institute of Biochemistry and Cell Biology, Chinese Academy of Sciences. All cells were maintained in RPMI-1640 medium (Life Technologies), supplemented with 10% fetal bovine serum (Life Technologies) and antibiotics (penicillin-streptomycin) under optimal conditions at 37 °C in a humidified atmosphere containing 5% CO_2_. Notably, the authenticity of OVCAR-3 and SK-OV-3 cell lines was verified through short tandem repeat profiling, as detailed in the supplementary material short tandem repeat certification report.

### IHC staining

The IHC protocol involved deparaffinization, antigen retrieval with citric acid buffer, and blocking to enhance antibody accessibility and specificity. The tissue sections were then incubated overnight with a primary ZNF280A antibody (1:200, Abcam ab169117) at 4 °C. Following rinsing with PBS, a secondary IgG antibody (1:400, Abcam ab6721) was applied for 3 h at room temperature. Visualization was achieved through 3,3'-diaminobenzidine (DAB) chromogenic staining and hematoxylin counterstaining. The IHC score was assessed using a literature-based method (29), with ZNF280A expression determined by its median in OC tissue.

### Plasmid construction and OC cell transfection

The lentiviral vectors harboring short hairpin RNA (shRNA) specifically targeting ZNF280A or ACRV1, as well as a ZNF280A-overexpressing lentivirus, were crafted by Shanghai Biosciences. In addition, a scramble shRNA vector shCtrl served as negative controls in our experiments. The shRNA sequences were as follows: shZNF280A-1: 5′-CTGTCACTATGAAGTCTTCAT-3′; shZNF280A-2: 5′-TTGTGTAAGAAAGTGGAATCA-3′; shZNF280A-3: 5′-GCCGGAGCAACTGCAAGGGTT-3′; shACRV1-1: 5′- GCACATCTACAGGCACAAT -3′; shACRV1-2: 5′- GGATCTGCCAGAGGAACAT -3′; shACRV1-3: 5′-GCTACACATGTGCTTATAT-3′; shCtrl: 5′-TTCTCCGAACGTGTCACGT-3′.

The RNA interference sequences were meticulously inserted into either the BR-V-108 or LV-004 lentiviral backbone, both sourced from Shanghai Bioscience Co, Ltd, adhering strictly to the manufacturer's guidelines. After a 48-h incubation period, virus particles were meticulously harvested. Subsequently, these recombinant lentiviral plasmids were efficiently introduced into OVCAR-3 and SK-OV-3 cell lines utilizing Lipofectamine 3000, adhering to the manufacturer's protocol for optimal transfection efficiency.

### Quantitative real-time-PCR (qRT-PCR)

Upon reaching 80 to 90% confluency, cells were rinsed with PBS and harvested using trypsin-EDTA. Total RNA was extracted from the cells using the Trizol reagent (Thermo Fisher Scientific, Cat. # 204211) according to the manufacturer's instructions. The integrity and purity of the extracted RNA were assessed using a Nanodrop 2000/2000C spectrophotometer (Thermo Fisher Scientific). Reverse transcription was performed to convert the extracted RNA into complementary DNA using a high-fidelity reverse transcriptase kit (Promega M-MLV Reverse Transcriptase). Next, qRT-PCR was performed in a mixture containing synthetic complementary DNA, a ZNF280A gene-specific primer and a caretaker gene (GAPDH as a reference) and containing a fluorescent reporter dye (SYBR Green) at a suitable temperature (primer annealing 25 °C, reverse transcription 42 °C, and enzyme inactivation 70 °C). The relative expression levels of ZNF280A were calculated as 2ˆ^(-ΔΔCt)^. The primer sequence as follows: ZNF280A, forward primers: 5′-GATCTGATCTATGTTGGGGTGGA-3′ and reverse primers: 5′-CGTGAGCAGGATATTGACGGA-3′; ACRV1, forward primers: 5′-TTCAAGCACATCTACAGGCACAA-3′ and reverse primers: 5′- GTTCCCTCTCCACGAAGACATTT-3′; CUX1, forward primers: 5′- ACAGCAGCTGCAGAGAGAAC-3′ and reverse primers: 5′- CTTGAACTCCCGGCTCTGTT -3′; CUX2, forward primers: 5′- GATCTACGGCGACTCCAGAAG-3′ and reverse primers: 5′- TTCCCGGCGGAGTTCAATTA -3′; GAPDH, forward primers: 5′- TGACTTCAACAGCGACACCCA-3′ and reverse primers: 5′- CACCCTGTTGCTGTAGCCAAA-3′; ACRV1 promoter site 01, forward primers: 5′- GCTTGGATCTGCCAGAGGAA-3′ and reverse primers: 5′- GCCTCAGCATCAGAAGGGTT -3′; ACRV1 promoter site 02, forward primers: 5′- AACCCTTCTGATGCTGAGGC-3′ and reverse primers: 5′- TTCTCCAGAAGTGTGCTCGG -3′; ACRV1 promoter site 03, forward primers: 5′- GCATGGTTCAAGCAAGCACA-3′ and reverse primers: 5′- CCTTCAGCATGTTCAGTCGC -3′.

### Western blotting and Co-IP

Total protein lysates were prepared from cell lines using the BCA Protein Assay Kit (Thermo Fisher Scientific, Cat. # A53227) according to the manufacturer's protocol. Equal amounts of protein (typically 20–50 μg per lane) were mixed with SDS-PAGE sample buffer, loaded onto a 10% SDS-polyacrylamide gel and subjected to electrophoresis at a constant voltage. After that, the proteins were transferred from the SDS-PAGE gel onto a polyvinylidene difluoride (PVDF) membrane using a wet transfer system (Bio-Rad Trans-Blot Turbo Transfer System). The membranes were blocked and probed with primary antibodies specific to the targets (anti-ZNF280A, 1:1000, Abcam, Cat. # ab169117), (anti-ACRV1, 1:500, Santa Cruz, Cat. # sc-398536), (anti-CUX1, 1:2000, Proteintech,Cat. # 11733-1-AP), (anti-CUX2, 1:500, Omnimabs, Cat. # OM631845) and GAPDH (1:3000, Bioworld, Cat. # AP0063) as a control. After washing, a secondary antibody IgG (Goat Anti-Rabbit, 1:3000, Beyotime, Cat. # A0208) was applied, and immunoreactions were visualized using ECL + plusTM. The resulting chemiluminescent signal was captured and analyzed to quantify protein expression, with GAPDH normalization ensuring accurate comparison between samples. For Co-IP, cell lysates were incubated with antibodies at 4 °C overnight, followed by incubation with protein A/G beads at 4 °C for 2 h. The beads were washed with radio-Immunoprecipitation assay (RIPA) buffer and boiled in SDS buffer for 10 min. The eluent was analyzed by western blotting.

### Colony formation assay

OVCAR-3 and SK-OV-3 cells were digested with trypsin, resuspended, counted, and inoculated on 6-well plates with 800 cells per well. Next, they were incubated for 10 days t to allow colonies to form, with the mediums being replaced every 3 days. After that, the cells were washed with PBS, fixed with paraformaldehyde for 1 h, stained with Giemsa for 20 min, washed three times with ddH_2_O, and finally photographed using a digital camera. The number of colonies (>50 cells per colony) was then counted under fluorescence microscopy (MicroPublisher 3.3RTV; Olympus). Colony formation efficiency (%) = (Number of colonies/Plated cells) × 100. Unpaired *t* test with Welch’s correction (*p* < 0.05).

### CCK-8 assay

OVCAR-3 and SK-OV-3 cells were seeded into 96-well plates at a density of 1000 cells per well in 100 μl of RPMI-1640 medium supplemented with 10% fetal bovine serum and 1% penicillin-streptomycin. Cells were added to respective wells in triplicate and allowed to adhere for 24 h at 37 °C in a 5% CO_2_ incubator. At that, 10 μl of CCK-8 reagent (DoJindo Laboratories) was added to each well, bringing the total volume to 110 μl. Plates were incubated at 37 °C for 2 h under standard culture conditions. To minimize edge effects, the outermost wells of the 96-well plate were filled with 200 μl of PBS to prevent evaporation. Absorbance at 450 nm (*A*_450_) was measured using a microplate reader (FlexStation 3, Molecular Devices) with background subtraction at 650 nm. Each experiment was performed in triplicate, and data were normalized to the negative control group. Proliferation multiples were calculated as: (*A*_450_ (treated) − I_450_ (0h))/(A_450_ (Control)−I_450_ (0h)). Statistical significance was determined by two-way ANOVA with Šídák’s multiple comparisons test (*p* < 0.05).

### Flow cytometry apoptotic assay

OVCAR-3 and SK-OV-3 cell monolayers were gently trypsinized to release adherent cells, which were then thoroughly resuspended in a suitable buffer to maintain cell integrity. The cell suspension was aliquoted into flow cytometry tubes, and Annexin V-APC and propidium iodide (PI) dyes were added according to the manufacturer's protocol. Following staining, the samples were promptly analyzed using a flow cytometer equipped with appropriate laser and filter settings to detect APC and PI fluorescence. The acquired data were gated to exclude debris and doublets, and the percentages of cells in different phases of apoptosis (early apoptotic: Annexin V-APC+/PI-, late apoptotic: Annexin V-APC+/PI+, and necrotic: Annexin V-APC-/PI+) were calculated using the software accompanying the flow cytometer. Apoptotic rate (%) = (Early apoptosis + Late apoptosis)/Total cells × 100. One-way ANOVA with Tukey’s *post hoc* test (*p* < 0.05).

### Transwell assay

OVCAR-3 and SK-OV-3 cells were seeded in the upper chamber of a Transwell insert precoated with matrix glue, where they were cultured in 100 μl of serum-free medium to promote a migratory stimulus. The lower chamber, separated by a permeable membrane, contained 500 μl of medium supplemented with a high concentration of serum, serving as a chemoattractant to induce cell migration. After an incubation period of 24 h, allowing for sufficient time for migratory cells to traverse the membrane, the Transwell insert was carefully removed, and the membrane was examined for the presence of migratory cells adhered to its underside. These cells were subsequently fixed with methanol for 30 min and stained with 0.1% crystal violet solution for 20 min. The membrane was gently rinsed with water to remove excess stain, photographed, and counted under a microscope.

### Wound-healing assay

Briefly, OVCAR-3 and SK-OV-3 cells were seeded into 96-well plates and allowed to adhere for 24 h at 37 °C in a humidified atmosphere containing 5% CO_2_. Following adhesion, cells were serum-starved to minimize proliferation-driven migration and ensure that observed migration was due to cell motility. At a predefined time point, a sterile scratch was introduced at the center of the well bottom using a scratch meter, pushing gently and uniformly upwards to create a clear, defined gap. Images of the scratched area were captured immediately after wounding (time 0) and at subsequent time intervals. Cell mobility was quantified by measuring the closure of the scratch over time, calculating the migration distance between the initial and subsequent time points using appropriate image analysis software.

### Glycolysis assay

OVCAR-3 and SK-OV-3 cells were seeded into 96-well plates and further cultured in a humidified atmosphere containing 5% CO_2_. The contents of glucose, lactic acid, and ATP were measured according to the instructions of the assay kit (Beijing Solarbio Science & Technology Co, Ltd). The extracellular acidification rates were measured using XFe96 Extracellular Flux Analyzer (Agilent) and BCA (Pierce) according to manufacturer’s instructions.

### Animal xenograft model

The animal study was conducted in accordance with the ethical guidelines approved by the Institutional Animal Care and Use Committee of Army Medical University. BALB/c female nude mice (4 weeks old) were procured from Beijing Wei Tong Li Hua Experimental Animal Technology Co, Ltd and housed under standard laboratory conditions. SK-OV-3 cells were thoroughly digested using trypsin and resuspended in an appropriate medium. Subsequently, mice were randomly assigned to two groups: the negative control group (shCtrl) and the experimental group (shZNF280A). Each mouse in both groups received a subcutaneous injection of 200 μl of SK-OV-3 cells suspended in PBS into the right forelimb axilla. Post injection, mice were monitored closely, with body weight and tumor size measured every 2 days for a week to ensure animal welfare and track tumor growth. After 22 days, mice were humanely euthanized, and tumors were excised, weighed, and photographed for documentation.

### Statistical analysis

The mean ± SD (n ≥ 3) was used to express the data, and the analysis was conducted using GraphPad Prism 8.0 software (GraphPad Software Inc). T-tests were utilized for comparing the difference. Any *p* values below 0.05 were regarded as statistically significant.

## Data availability

The data generated/analyzed during the current study are available.

## Supporting information

This article contains supporting information.

## Conflict of interests

The authors declare that they have no conflicts of interest with the contents of this article.
