# Supplementary material for: ZNF280A and ACRV1 enhance aerobic glycolysis and drive ovarian cancer progression via the PI3K/AKT signaling pathway
Source: J Biol Chem. 2025 Dec 1;302(1):110993. doi: 10.1016/j.jbc.2025.110993 (PMC12803836; doi:10.1016/j.jbc.2025.110993)
Supplement: Supplementary Material 1 [file mmc1.docx]

**
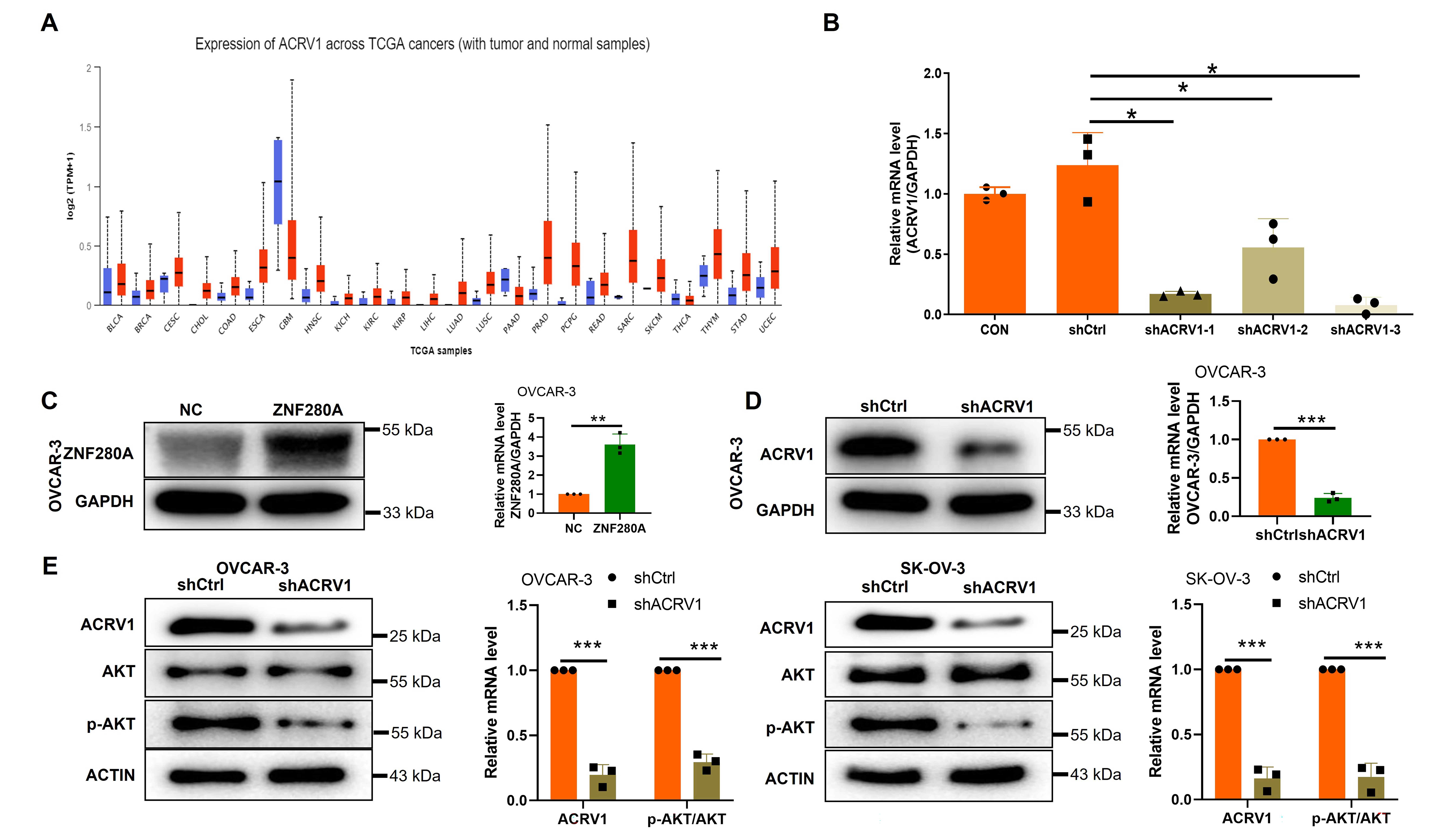
**

**Supplementary Fig S1 The expression of ZNF280A and ACRV1 in cancers and their effects on migration and PI3K/AKT.**

**(A)** TCGA online database analysis showed that ACRV1 expression was abnormally upregulated in various cancers. **(B)** Three small hairpin RNA (shRNA) interference ACRV1 sequences were designed and screening the sequence with the highest knockdown rate by RT-PCR. **(C-D)** Overexpression of ZNF280A and ACRV1 knockdown were present in A2780 cells and verified by WB. **(E)** The expression of PI3K/AKT signaling pathway-related proteins was detected by western blot assay in ACRV1 knockdown A2780 cells.
